# Medical ethics and compliance amongst physician groups: a self-assessed survey in a hospital in Southeast Nigeria

**DOI:** 10.4314/ahs.v23i3.84

**Published:** 2023-09

**Authors:** Ogbonnia Godfrey Ochonma, Udunma Olive Chjioke, Justin Agorye Ingwu, Chikezie Adolf Nwankwor, Ifeyinwa Henry-Arize

**Affiliations:** 1 Department of Health administration and Management, Faculty of Health Sciences and Technology, University of Nigeria, Enugu Campus, Enugu State, Nigeria; 2 Department of Business Administration, Coal City University, Enugu, Enugu State, Nigeria; 3 Department of Nursing, Faculty of Health Sciences, College of Medicine, University of Nigeria, Enugu Campus - Nigeria

**Keywords:** Professional, ethical, conduct, doctors, patients, survey, Nigeria, hospital

## Abstract

**Background:**

Being a doctor remains a moral enterprise as he is expected to make some medical decisions based on ethical principles during encounter with patients.

**Objective:**

The objective of this study was to investigate the knowledge and application of medical ethical principles amongst physician groups in a Hospital in Enugu, Nigeria.

**Methods:**

This was a cross-sectional self-assessed study conducted amongst medical doctors in five specialty groups in a teaching hospital in Enugu, Nigeria.

Descriptive and inferential statistics were used to summarize the items and determine whether significant differences on knowledge and application of medical ethics existed amongst the physician groups in the treatment of patients.

**Findings:**

Observance and compliance with medical ethical conduct was highest among doctors that were aged 55 years and above. In sex, male doctors had higher ethical conduct compliance than female doctors. Comparing the doctors by rank, medical officers, consultants and senior registrars respectively had the highest ethical conduct.

**Conclusions:**

Knowledge and practice of medical ethics were mostly deficient among younger Nigerian and female doctors. Remedying the situation will require better curricula both at the undergraduate and post-graduate medical school programmes for doctor trainees. Requiring certification in bioethics for license renewal will also help in resolving and improving the knowledge gap.

## Introduction

The doctor–patient relationship is critical for vulnerable patients and this is usually because patients do overly rely on the physician' competence, skills, and good will for their medical treatment 1, 2. United States law considers the doctor–patient relationship as fiduciary as physicians are expected and required to act in the best interest of their patients even when that interest conflicts with theirs 1, 3. Also, the doctor–patient relationship is noted for its centrality in the patient's life-altering treatments, births, deaths and severe illnesses 4.

Codes of ethics remain the professional control of the behaviour of doctors as it represents a commitment to act with integrity even in extreme circumstances 5 6. Ethical codes of conduct have offered both patients and doctors some tangible protection in some circumstances^5^,^6^. Most ethical codes are concerned with attitudes and expected forms of conduct, for example providers are expected to act in the best interest of their patients and delivering bad news with understanding and sympathy 5 7. The ethical principles and codes are most notably described by Beauchamp and Childress 5 8, which is based on four principles: autonomy, non-maleficence, beneficence, and justice. The principles and codes are explained to justify one another at different levels. Both the methodology and applicability 5 9 of principlism 5 10 have been challenged, as well as defended as a common framework for biomedical ethics 5 11, 12.

All health care practitioners are obliged to uphold the best interest of patients by the principles of professionalism which embodies the concept of fiduciary relationship. The law sets the minimum standards of conduct which must be obliged by the professionals 13, 14 in patient care.

Modern thought on beneficence which embraces humanism emphasises that all persons have immutable rights to life and liberty that must be respected and nurtured 13, 15, 16. Practitioners must act in the best interest of the patient and refrain from harming him 17. Beneficence stresses for the best possible care and avoidance of harm to the patient 18. The ethic of non-malfeasance has an obvious relationship with beneficence in that it equally emphasises the avoidance of any act that would cause harm to the patient 18.

The principle of Autonomy concerns the duty to respect individuals' right to choose which health care interventions are acceptable to them but excludes unsafe options 19. Related to autonomy is the patient's choice of confidentiality which requires providers to keep patient's information confidential unless the patient consents to release^20^.

Justice on the other hand addresses what entitlements are due to individuals for their health care. Individual's right to fair and equitable distribution of the benefits and the risks or burdens of available health care is emphasised by this principle 19. This principle also requires fair distribution of health outcomes to achieve equity 21. In a very prominent conception of justice in the context of health, Daniels 21, 22 considers health equity as a matter of fairness and justice.

There is paucity of researched information on medical ethical conduct, however, physicians are expected to be respectful, courteous and act in a civil manner towards clients and colleagues 23. Engaging in these actions will promote trust, shared accountability and collaboration 23. On the other hand, behaviour that shows unprofessional and/or disruptive tendency undermines medical professionalism and the trust of the public 24, 25, 26. These behaviours can erode physician/patient communication which is at the centre of good medical practice 24, 25, 26, 27. There are limited studies exploring physicians' professional/ethical behaviours in the process of patient treatment and care especially in the African context. This study investigated the knowledge and application of medical ethical principles amongst physician groups in a teaching hospital in Enugu, Nigeria.

## Methods

### Study Area

The study area for this project is located within Enugu metropolis in Enugu state, South-east Nigeria. Enugu is host to three major health institutions. The state is among the 36 Federating states of the Federal Republic of Nigeria. The population of the state hovers around 3.3 million people (2006 census). Ninety five (95%) of the population are of the Igbo tribe/extraction. Above half (59%) of the population are rural dwellers. The State is made up of 17 Local Government Areas (LGAs) with 3 senatorial zones for administrative purposes comprising Enugu North, Enugu East and Enugu West Senatorial zones (SMOH, Enugu State, 2001). The study was conducted in one of the government-owned teaching hospitals in Enugu.

### Study design, Sample population and Sample size

The government hospital where this work was carried out comprised of two hundred and three (203) medical doctors as at the time the study was carried out. All the doctors were included in the study however one hundred and forty (140) doctors were able to fill and return their questionnaires giving a response rate of 68.9%.

### Instrument validation and Pre-test

The instrument was face validated by three senior faculty members in the Faculty of Health Sciences and Technology, University of Nigeria, Enugu Campus. The questionnaire was first developed and pilot-tested with similar respondents (physician groups) from a different hospital to measure their understanding and relevance of the contents of the questionnaire. The respondents made their comments as to how the contents of the questionnaire could be enhanced to measure the objective of the study which is to investigate how much doctors knew and applied medical ethical principles in the treatment of patients. The results of the pilot-test were analysed, modified and included in the questionnaire and passed on to senior colleagues who endorsed and approved of the final questionnaire. The Cronbach alpha before the questionnaire was modified was .675

### Ethical approval

Ethical approval for this study was obtained from a local ethics review committee (University of Nigeria ethics review committee).

### Data Analysis

Both descriptive and inferential statistics were used in the analysis of this work. The descriptive statistics- frequency, percentage, mean and standard deviation were used to summarize the items depending on whether the data is nominal, ordinal or numerical. The inferential statistics-- Independent Samples t-test and One Way Analysis of Variance (ANOVA) for between-groups design were used to determine whether significant difference existed between groups that were compared. The Duncan Multiple Range Test served as a Post Hoc test for the ANOVA. The Likert-scaled items were used to generate an ethical conduct score which served as the dependent variable used for the t-test and ANOVA. Normality and equality of variance assumptions was assured before the statistics were used. A logistic regression was also performed on the data. The demographic data served as the predictors while the ethical conduct score was categorised to binary variable which served a predicted variable.

Preliminary analysis indicated that most of the doctors were aged between 25-44 years (87.9%). Males (60.7%) were more than females (39.3%). Majority of the doctors were house officers (40.0%) while consultants were the least (5.7%). Their areas of specialty were community health (22.9%), surgery (16.4%), internal medicine (22.1%), paediatrics (20.0%) and obstetrics and gynaecology (18.6%). Almost all knew code of medical ethics in their undergraduate medical school (91.4%). Those who knew that National Health Research Ethics Committee supervises all medical ethics issues were fairly above average (58.6%).

In assessing the ethical conduct of doctors, among the eight of most observed ethics by the doctors were: improvement of competence to serve patients better (3.97±0.17), observance of the principle of confidentiality of information acquired through professional contact with patients (3.68±0.60), building and sustaining professional relationships as both an independent practitioner and collaborative member of a team (3.04±1.17). The least of the observed ethics was: exercising the need to engage patients in planning and evaluating diagnostics, treatments and interventions to meet their health needs and goals (1.36±0.73).

Ethical conduct and compliance among doctors were highest among doctors that were aged 55 years and above (44.00±4.97) and was least among doctors who were aged 45-54 years. Comparison between the age groups, using ANOVA for independent groups, revealed no significant difference, F (3, 132) = .362, p = .780. This implies that ethical conduct and compliance of doctors was almost the same for the four age groups.

However, there was a significant difference in the ethical conduct and compliance of doctors when classified by their area of specialty, F (4, 131) = 6.272, p < .001. A Post Hoc test using Tukey's HSD revealed that doctors that specialised in community health (46.23±4.25) had ethical conduct significantly higher than others, p < .05 while others (surgery, internal medicine, paediatrics and obstetrics and gynaecology) were the same, p = .746.

In knowledge of supervising body of all medical issues in Nigeria, those that have correct knowledge (43.56±4.95) had more ethical conduct than those with incorrect knowledge (42.00±3.99). This difference between them, as revealed by Independent Samples t-test for unequal variances, was significant, t (128.27) = 2.013, p = .046.

Ethical conduct and compliance scores of doctors revealed that greater part of them had good ethical conduct 95(67.9%). That is, their ethical conduct was assessed above average, while the remaining 41(29.3%) had ethical conduct compliance assessed below average.

The logistic regression model explained 27.6% (Nagelkerke R^2^) of the variation in doctors' ethical conduct status (that is, whether good or poor). It also correctly predicted the status of 74.1% doctors. The omnibus test of model coefficients using the Chi-Square revealed that the model coefficients were significant, χ^2^ (13) = 29.260, p = .006. The Wald statistic further indicated that the coefficient of gender and area of specialty were significant, p < .05. Holding other predictors constant, male doctors had odds 3.3 times more than the female doctors in being classified as doctor with good ethical conduct with 95% C.I of 1.15, 9.69. Overall result indicates that community health doctors had odds 12.3 times, 12.0 times and 13.7 times the odds of surgeons, internal medicine doctors and paediatricians respectively in being classified as a doctor with good ethical conduct.

These statistical techniques were done using the IBM SPSS version 20.

## Results

Table 1. The socio-demographics of the participants displayed the characteristics of the doctors who were involved in the study. This included the age groups and gender of the doctor participants, their specialties, present rank, and knowledge source of medical ethical code including knowledge of the supervising body of all medical ethical issues.

**Table 1:** Socio-demographic data of the participants n=140

	Groups	Frequency	Percent
Age	25-34 years	69	49.3
	35-44 years	54	38.6
	45-54 years	10	7.1
	55+ years	7	5.0
		Total:140	100%
Gender	Male	85	60.7
	Female	55	39.3
		Total: 140	100%
Present rank	House officer	56	40.0
	Medical officer	21	15.0
	Registrar	40	28.6
	Senior registrar	15	10.7
	Consultant	8	5.7
		Total:140	100%
Area of specialty	Community health	32	22.9
	Surgery	23	16.4
	Internal medicine	31	22.1
	Paediatrics	28	20.0
	Obstetrics & Gynaecology	26	18.6
		Total:140	100%
Knowledge source of code of medical ethics	Undergraduate medical school	128	91.4
	Internet and medical journals	12	8.6
	Continuous medical education	0	0.0
	Extra courses	0	0.0
	Post graduate medical school	0	0.0
		Total:140	100%
Knowledge of the supervising body of all medical ethics issues	Correct (National Health Research Ethics Committee)	82	58.6
	Wrong	57	40.7
		Total:139	99.3

Missing data exists in item if total frequency is less than 140

Table 2. The assessment of medical professional/ethical conduct of doctors displays the knowledge the doctors have concerning medical ethical issues and their applications during patient encounter. This exercise was done using a 4 (four) point Likert scale as follow: A = Agree, MA = Moderately agree, D = Disagree, HD = Highly disagree for each of the questions.

**Table 2:** Assessment of medical professional/ethical conduct of doctors n =140

	A (4)	MA (3)	D (2)	HD (1)	M±SD
Have you always honoured the principle of informed consent including the right of patients or their substitute to refuse service observed?	16	15	34	75	1.80±1.03
Have you always observed the dignity, privacy and autonomy of patients during their treatment?	29	36	36	37	2.41±1.10
Have you always maintained appropriate professional boundaries during services provision to your patients?	60	29	36	15	2.96±1.06*
Have your patients been treated equitably regardless of their tribe, ability to pay and type of illness?	42	61	32	5	3.00±0.82*
Have you always provided individualized service to your patients during examination taking into account their particular physical and emotional needs, values and background?	16	17	37	70	1.85±1.03
Have you always observed the principle of confidentiality of information acquired through professional contact with your patients?	103	30	4	2	3.68±0.60*
Have you always completed your patients' examination on timely manner and in response to their needs?	59	31	31	19	2.93±1.09*
Do you normally explain your experience, expectations, knowledge and equipment procedure to your patients before service to improve patients' confidence?	54	27	28	31	2.74±1.19*
Have you always improved on your competence to serve your patients better?	136	4	0	0	3.97±0.17*
Is it always in your habit to take personal responsibility, use discretion and judgment in a manner that ensured the best service outcome to your patients?	56	32	41	11	2.95±1.01*
Have you always disclosed your competence and limitations where appropriate in the process of service provision to assure patients' confidence?	8	12	18	102	1.47±0.88
Have you always acted in the best interest of your patients during contact and service delivery?	59	31	25	25	2.89±1.14*
Have you always built and sustained professional relationships as both as an independent practitioner and collaboratively as a member of a team?	74	21	21	24	3.04±1.17*
Have you always exercised the need to engage your patients in planning and evaluating diagnostics, treatments and interventions to meet their health needs and goals?	4	9	20	106	1.36±0.73
Have you always been able to make appropriate referrals as the need be in the process of care and examination?	57	46	16	21	2.99±1.06*
Have you always demonstrated effective and appropriate skills in communicating, advice, instructions and professional options to your patients and as the case may be their relatives?	56	22	38	24	2.79±1.15*

Item with M > 2.5 were considered as ethical conducts observed by doctors

* indicates conducts observed by doctors (M > 2.5)

Missing data exists in item if total frequency is less than 140; A = Agree, MA = Moderately agree, D = Disagree, HD = Highly disagree

Table 3. Amongst the doctors, there existed variations in professional/ethical conduct as known and complied with during patient encounter according to their socio-demographics as in age, sex, rank, specialty and was precisely captured in the analysis and displayed in the table.

**Table 3:** Variations in professional/ethical conduct of doctors

	Groups	n	M±SD	df	t	F	p-value
Age	25-34 years	67	42.84±4.93	3, 132	-	.362	.780
	35-44 years	52	43.13±4.53				
	45-54 years	10	41.80±2.66				
	55+ years	7	44.00±4.97				
Sex	Male	82	43.40±4.85	134	1.463	-	.146
	Female	54	42.22±4.20				
Rank	House officer	55	42.69±5.23	4, 131	-	.595	.667
	Medical officer	20	44.10±3.04				
	Registrar	38	42.34±4.66				
	Senior registrar	15	43.33±4.01				
	Consultant	8	43.75±4.65				
Area of specialty	Community health ^a^	31	46.23±4.25	4, 131	-	6.272	< .001
	Surgery ^b^	22	42.23±4.98				
	Internal medicine ^b^	31	41.81±4.98				
	Paediatrics ^b^	28	41.29±3.90				
	Obstetrics & Gynaecology ^b^	24	42.71±3.00				
Knowledge source of code of medical ethics	Undergraduate medical school Internet and medical journals	124	42.97±4.68	134	.274	-	.784
		12	42.58±4.14				
*Knowledge of supervising body of all medical ethical issues	Correct	81	43.56±4.95	128.265	2.013	-	.046
	Incorrect	54	42.00±3.99				

Analysis excluded some participants due to missing data

* t-test computed for unequal variance

Table 4. Overall ethical conduct status classification of the doctors shows how the doctors fared in numbers and percentages either as poor or good ethical compliant.

**Table 4:** Overall Ethical Conduct Status Classification

		Frequency	Percent
Status	Poor (overall ethical conduct score < mean)	41	29.3
	Good (overall ethical conduct score > mean)	95	67.9

Mean of overall ethical conduct score = 40; Excluded 4 (2.9%) participants due to missing data

Table 5. Shows how the characteristics of the doctors predicted who would have good ethical conduct.

**Table 5:** Logistic Regression Model Coefficients

Predictors	OR	p-value	95% C.I. for OR	
			Lower	Upper
Age		.874		
- 25-34 years	.338	.642	.003	32.788
- 35-44 years	.446	.617	.019	10.564
Gender (male)	3.267	.027	1.141	9.357
Rank		.144		
- Medical officer	6.360	.100	.703	57.571
- Registrar	.562	.729	.022	14.574
- Senior registrar	1.025	.991	.017	60.301
- Consultant	.440	.745	.003	62.779
Area of specialty		.009		
- Surgery	.081	.004	.015	.452
- Internal medicine	.083	.002	.017	.416
- Paediatrics	.073	.001	.015	.349
- Obstetrics & Gynaecology	.293	.166	.051	1.666
Knowledge source of medical ethics	1.608	.556	.331	7.820
Knowledge of supervising body (Correct)	1.255	.611	.523	3.008
Constant	15.477	.264		

Nagelkerke R^2^ = .276, Omnibus Test of Model Coefficient: χ^2^ (13) = 29.260, p = .006; Status correctly predicted = 74.1%

Predictor reference category: Age (45+ years), Gender (Female), Rank (House officer), Specialty (Community medicine), Knowledge source of medical ethics (undergraduate medical school); knowledge of supervising body of all medical ethical issues (wrong)

## Discussion

This study noted that the most observed areas in medical ethical treatment of patients by physicians were within the areas that affected patients' treatment the most like improvement of competence to serve patients better, observance of the principle of confidentiality of information, building and sustaining professional relationships, treating patients equitably, making appropriate referrals as need be in the process of patient care and examination. These behaviours promote the mandates as contained in the codes and principles of patient care and are very much supported by Kisinger^13^ and Leape et al 24 who stated that physicians are expected to take responsibility for their behaviours and to meet the obligations and expectations set out for them along with the expectations set out in institutional codes of conduct, policies or by-laws. The institutional codes of conduct set the minimum standards of conduct which must be obliged to by doctors in the process of patient encounter. These codes of medical ethics remain the professional control of the behaviour of doctors as it represents a commitment to act with integrity even in extreme circumstances.

Ethical codes have the potential of offering both patients and doctors some tangible protection in some circumstances regarding medical malpractice. Doctors who are able to show that they confined their behaviour to these specified codes of conduct during medical patient engagement, could be exonerated from liability even when patients try to prove medical misconduct on the part of the doctor. So, being guided by institutional codes of conduct works in protection of patients who receive maximum care and also the physician who avoids medical liability.

The least four ethical behaviours observed by the physicians were: exercising the need to engage patients in planning and evaluating diagnostics, disclosing their competence and limitations where appropriate in the process of service to assure patients' confidence, honouring the principle of informed consent and providing individualized service to patients during examination. These behaviours as exhibited by the physician are very disruptive and undermine the expectations of the patients and society at large and very much in negation with 27 where it was stated that physicians must not engage in disruptive behaviours because they undermine professionalism as well as a culture of safety.

The results of the study indicated that almost all the doctors knew about code of medical ethics. But this high level of knowledge was not reflected in actual practice as many doctors still lacked in some basic behavioural ethical conduct like honouring the principle of informed consent and providing individualized service to patients during examination for example. Doctors are relied upon by patients and as such required to seek the consent of their patients in all medical procedures as patients are presumed to lack basic medical knowledge on procedures. Non-adherence to this ethical principle not only violet the patient's dignity as a person, but could as well lead to potential law suit when such procedure results in medical misconduct or liability on the part of the doctor.

There was a significant difference in the ethical conduct of doctors classified by their area of specialty. Doctors that specialised in community health had ethical conduct significantly higher than others. There was significant difference in knowledge of medical ethical practice when doctors were compared based on age groups. As expected, doctors of the highest age group (55 and above) scored the highest in that category by indicating having observed and tried dealing with ethical issues when patients were being treated in hospitals. This would have come from their continued experience and exposure in the field. Reasonable measures must ensure equity in knowledge of ethical conduct of doctors to discourage the disparity in knowledge as witnessed amongst the doctors. Medical ethics curriculum should be modified and introduced to all specialties of the medical profession reflecting the need for improved and applied knowledge of medical ethics. Seminars, workshops and conferences on medical ethics should be enhanced within the medical profession and amongst all the specialties to improve knowledge. Case studies bordering on medical ethics should be taught to younger doctors by qualified and certified bioethicists who should emphasize its application in modern medicine to bridge the gap in knowledge heavily in favour of older doctors. Certification in medical ethics should be made a requirement for medical license renewal amongst the younger and female doctors to further bridge the gap in knowledge in favour of older doctors.

## Conclusion

The most observed knowledge of medical ethics by the doctors were: improvement of competence to serve patients better, observance of the principle of confidentiality of information acquired through professional contact with patients and building and sustaining professional relationships as both an independent practitioner and collaborative member of a team. The least observed and applied medical ethical principle being exercising the need to engage patients in planning and evaluating diagnostics. On the whole, ethical conduct and compliance scores of doctors revealed that greater part of the doctors had good knowledge and compliance with medical ethics in the treatment of patients.

Reducing the gaps in knowledge of medical ethics amongst physician groups, requires more emphases on reorganising the curricular at both the undergraduate and post-graduate medical schools.

Sharing ethical conduct experiences especially between older and younger doctors will be helpful in bridging the knowledge gaps as well. Ways should be devised for female doctors to gain more ethical conduct experiences through sharing of information and supervision by medically more experienced older male doctors.

Requiring certification in bioethics for medical license renewal will also help in resolving the knowledge gaps especially for the younger and female doctors.

## Strength and limitation of the study

This study has explored in general the knowledge base of medical doctors in a Nigerian tertiary institution concerning medical ethics and compliance. It gathers its strength from the fact that doctors of different specialties were involved in the study. The limitation of this study is that only one institution was involved. Future studies should improve on this by including more institutions.

## Citation

Ochonma OG; Chijioke UO; Ingwu JA; Nwankwor CA; Henry-Arize I. Medical Ethics and Compliance amongst Physician Groups: A Self-assessed Survey in a Hospital in Southeast Nigeria. African Health Sciences.
